# Retraction: Determining the appropriate level of farmyard manure biochar application in saline soils for three selected farm tree species

**DOI:** 10.1371/journal.pone.0325643

**Published:** 2025-06-04

**Authors:** The PLOS One Editors

The *PLOS One* Editors retract this article [1] because it was identified as one of a series of submissions for which we have concerns about authorship, competing interests, and peer review.

In reviewing this matter, we noted that Tables 1-3 and 5 in [1] report results previously published in [2]. The prior article [2] was cited as Reference 36 in [1], but the reuse of results was not acknowledged.

In addition, the following concerns call into question the reliability of results reported in Table 5:

◦ FYMB 6% Sp. 1 and Sp. 2 report the same results.
◦ EC (dS/m) = 10 x TSS (mmol/L) in all cases.
◦ Within each row, the standard error is the same across all columns.
◦ There are repeating values across columns. For example, two of three species per treatment group have the same pH. Similarly, TOC levels are repeated across species.
◦ The means, but not the standard error values, for Control groups re-report findings published in Table 3 of [2]. However, the FYMB 6% results differ across the two articles.

The authors explained that [1] and [2] used data from the same PhD dissertation. They acknowledged that they did not disclose the data reuse in Tables 1-3 of [1]. Concerning the issues noted in Table 5, the authors stated that the table reports the experimental results and any similarities or repeat values may reflect the true experimental outcomes; they also offered to review the dataset to verify the accuracy of the data reporting. PLOS did not pursue this further because other unresolved issues summarized in the first paragraph of this notice support retraction independent of the data concerns.

MTBY, MFN, GY, SG, and AR did not agree with the retraction. HC, IA, MR, QX, and SUR either did not respond directly or could not be reached.

Tables 1–3 from [1] report material from [2], published in 2021 by Taylor & Francis, which are not offered under a CC BY license and are therefore excluded from this article’s [1] license.
